# Education and training in radiation protection in Europe: an analysis from the EURAMED rocc-n-roll project

**DOI:** 10.1186/s13244-022-01271-y

**Published:** 2022-09-04

**Authors:** Louise Rainford, Joana Santos, Francisco Alves, João Paulo Figueiredo, Christoph Hoeschen, John Damilakis, Guy Frija, Jonas Andersson, Jonathan McNulty, Shane Foley, Klaus Bacher, Ursula Nestle, Monika Hierath, Graciano Paulo

**Affiliations:** 1 Radiography and Diagnostic Imaging, School of Medicine, University College Dublin, Room A201, UCD Health Science Centre, Belfield Campus, Dublin 4, Ireland; 2 Instituto Politécnico de Coimbra, ESTESC - Coimbra Health School, Medical Imaging and Radiotherapy, Coimbra, Portugal; 3 Institute of Medical Technology, Otto-Von-Guericke University Magdeburg, Magdeburg, Germany; 4 School of Medicine, University of Crete, Crete, Greece; 5 Université de Paris, Paris, France; 6 Department of Radiation Sciences, Radiation Physics, Umeå, Sweden; 7 Division of Medical Physics, Department of Human Structure and Repair, Ghent University, Ghent, Belgium; 8 Department of Radiation Oncology, Kliniken Maria Hilf, Moenchengladbach, Germany; 9 Department of Radiation Oncology, University Hospital Freiburg, Freiburg im Breisgau, Germany; 10 European Institute for Biomedical Imaging Research (EIBIR), Vienna, Austria

**Keywords:** Radiation protection, Education, SWOT Analysis, Trainer development

## Abstract

**Background:**

A Strengths, weaknesses, opportunities and threats analysis was performed to understand the status quo of education and training in radiation protection (RP) and to develop a coordinated European approach to RP training needs based on stakeholder consensus and existing activities in the field. Fourteen team members represented six European professional societies, one European voluntary organisation, two international healthcare organisations and five professions, namely: Medical Physicists; Nuclear Medicine Physicians; Radiologists; Radiation Oncologists and Radiographers. Four subgroups analysed the “Strengths”, “Weaknesses”, “Opportunities” and “Threats” related to E&T in RP developed under previous European Union (EU) programmes and on the Guidelines on Radiation Protection Education and Training of Medical Professionals in the EU.

**Results:**

Consensus agreement identified four themes for strengths and opportunities, namely: (1) existing structures and training recommendations; (2) RP training needs assessment and education & training (E&T) model(s) development; (3) E&T dissemination, harmonisation, and accreditation; (4) financial supports. Weaknesses and Threats analysis identified two themes: (1) awareness and prioritisation at a national/global level and (2) awareness and prioritisation by healthcare professional groups and researchers.

**Conclusions:**

A lack of effective implementation of RP principles in daily practice was identified. EuRnR strategic planning needs to consider processes at European, national and local levels. Success is dependent upon efficient governance structures and expert leadership. Financial support is required to allow the stakeholder professional agencies to have sufficient resources to achieve a pan European radiation protection training network which is sustainable and accredited across multiple national domains.

## Key points

- Clinical, research and industry professionals administering ionising radiation must prioritise training.
- Employers and regulators must ensure radiation protection training is mandatory.
- Expert radiation protection trainers are needed for the European training network.
- Appropriate resourcing is required for radiation protection training development and implementation.

## Background

The application of ionising radiation and nuclear technologies play a crucial role in healthcare through the three main medical specialties radiology, radiotherapy and nuclear medicine. These three specialties are among the most innovative of all medical disciplines and early adopters of new technologies. The technological advances in these specialties have enabled significant progress in early detection and diagnosis, treatment selection and monitoring, image-guided intervention. Precise and normal tissue sparing curative treatment of oncological and non-oncological diseases have contributed significantly to developments in personalised medicine. This significant technological progress brings however new challenges, that society must be aware of, in particular the increased exposure of patients and staff to ionising radiation.

To find solutions for these challenges, the European Commission (EU) funded a project [1] (EURAMED rocc-n-roll—EuRnR), to propose an integrated and coordinated European approach to research and innovation in medical applications of ionising radiation and related radiation protection (RP) based on stakeholder consensus and existing activities in the field. The overarching objective of EuRnR is to generate a European consensus on research needs and their priorities in medical radiation application and corresponding RP to optimise the use of ionising radiation in medicine and thereby improve its benefit to Europe’s patients. Under EuRnR, an Education and Training (E&T) framework for health professionals and researchers will be developed. The framework will be based on an analysis of the current E&T capabilities and what is required to support its successful integration into practice and to support further research following the EURAMED Strategic Research Agenda (SRA) and roadmap [2] implementation.

It is widely recognised that E&T in RP for health professionals is vital towards the development of a RP safety culture to protect patients and staff from the dangers arising from the exposure to ionising radiation. The International Commission on Radiological Protection (ICRP) supports that professionals involved directly in the use of ionising radiation should receive E&T in RP at the start of their career, and the education process should continue throughout their professional life as the collective knowledge of the subject develops [3]. However, due to the rapid development of medical techniques based on ionising radiation, there is a strong demand for new E&T models in medical RP. The major challenge is to address the variety of professions working on a daily basis with ionising radiation, but having different knowledge background and also different needs with respect to E&T. All of them, however, are working towards the same objective: patient and staff safety [2]. Also the Heads of the European Radiation Protection Authorities (HERCA) supports that E&T requirements for RP knowledge and skills should cover underpinning science, RP philosophy and principles, management, organisation and practical application techniques and knowledge and skills of applicable legislation and guidance [4]. It is therefore understandable that, considering all these important and relevant aspects, the European Commission has reinforced the importance of education, information and training in the field of medical exposure, in Article 18 of the Council Directive 2013/59 EURATOM. This lays down basic safety standards for protection against the dangers arising from exposure to ionising radiation, by requiring that European Member States *“ensure that practitioners and the individuals involved in the practical aspects of medical radiological procedures have adequate education, information and theoretical and practical training for the purpose of medical radiological practices, as well as relevant competence in radiation protection”* [5].

To understand the status quo of E&T in RP in Europe, the EuRnR project performed a SWOT (Strengths, Weaknesses, Opportunities and Threats) and TOWS analysis of the results/impact of E&T RP outputs developed under previous EU framework programmes [6–8].

## Methods

To perform the SWOT analysis, the project team working on this task was split into four groups to analyse the “strengths” (group 1), “weaknesses” (group 2), “opportunities” (group 3) and the “threats” (group 4) of each document related to E&T in RP developed under previous EU programmes [9–11] and on the Guidelines on Radiation Protection Education and Training of Medical Professionals in the European Union [12].

Following the first analysis by the four groups, that resulted from a brainstorming amongst the group members, the draft SWOT matrix (planning tool) was sent out for review and comments to a dedicated cross disciplinary expert panel, composed of members of the project advisory board and external experts. This expert panel, composed of 14 members, integrated representatives from five European Professional Societies (CIRSE—Cardiovascular and Interventional Radiological Society of Europe; EANM—European Association of Nuclear Medicine; EFOMP—European Federation of Organisations for Medical Physics; EFRS—European Federation of Radiographer Societies; ESR—European Society of Radiology; ESTRO—European Society for Radiotherapy and Oncology), one European Voluntary Organisation (HERCA), two International Organisations (WHO—World Health Organisations; IAEA—International Atomic Energy Agency) and five clinical experts (Medical Physicist Expert; Nuclear Medicine Physician; Radiation Oncologist; Radiographer).

After incorporating the comments from the panel, the final version of the SWOT matrix was approved, and a TOWS (Threats, Opportunities, Weaknesses, and Strengths) analysis (an action tool) was performed as a strategy to address the results of the initial SWOT investigation and to define future strategies. The TOWS analysis was carried out by the aforementioned four groups to define how to minimise the threats and weaknesses by maximising the opportunities and strengths [13], thus overcoming a criticism of a standalone SWOT analysis by exploring the relationships between categories and specified factors. The findings for strengths—opportunities—activities sections were reviewed by all the working group members and consensus agreement reached on four main themes, namely: [1] Existing structures and training recommendations; [2] RP training needs assessment & E&T model(s) development; [3] E&T dissemination/harmonisation/accreditation; [4] Financial supports (Table 1 and 2). Similarly, weaknesses—threats—activities sections were evaluated for main themes and two were identified: [1] Awareness and prioritisation at a nation/global level and [2] Awareness and prioritisation by healthcare professional groups and researchers (Table 3 & 4).

**Table 1:** Summary of the weaknesses identified by SWOT analysis

Weaknesses evidenced due to a lack of	
Hands-on training courses	Proper understanding of the importance of RP in medicine
Profession-specific training	Recognition and/or professional accreditation in some countries
Novel training methods e.g. blended learning incorporating simulation/online options; bite-sized learning	Proper and updated evidence-based E&T materials
Long duration courses with comprehensive coverage of RP topics	Guidance on how to best train for RP topics
Standards on how to “train the trainers” to ensure that they can teach effectively	

## Results

### SWOT analysis of the results/impact of E&T in RP aspects developed under previous EU framework programmes and EU-funded projects

#### Strengths

Ten principal strengths were identified the following SWOT analysis, and the agreed text summarising the findings is as follows:

1. Ten-year history of collaboration across Europe via various radiation protection research platforms (MELODI—*Multidisciplinary European Low Dose Initiative*; EURADOS—*European Radiation Dosimetry Group;* and more recently EURAMED—*European Alliance for Medical Radiation Protection Research*) and research projects and partnerships (DoReMi—Low Dose Research towards Multidisciplinary Integration; OPERA—Open Project for the European Radiation Research Area; CONCERT—European Joint Programme for the Integration of Radiation Protection Research).
2. Recognised importance of E&T within EU project calls, with specific financial support to organise and manage E&T as part of EU-funded research projects.
3. Assessment of training needs already completed (ENETRAP; European Network on Education and Training in Radiological Protection; MEDRAPET—Medical Radiation Protection Education and Training).
4. Strategic research agendas of radiation protection platforms have been produced and disseminated and include E&T elements.
5. Existing guidelines for E&T in RP for health professionals (MEDRAPET).
6. Euratom regulation and National Competent Authorities in existence for many years.
7. Some continued financial support for E&T, even in initiatives not specifically targeting the medical field (e.g. ENEN +—European Nuclear Education Network).
8. Established Network and experience of organising European common training and initiatives on Education and Training in Radiological Protection in Europe (e.g. ENETRAP).
9. E&T initiatives support/encourage European mobility among students/trainees in the field of RP.

#### Weaknesses

A series of perceived deficiencies were identified and are summarised in Table 1. Two thirds of the weaknesses were in relation to training availability, training content and the training of trainers in radiation protection education.

#### Opportunities

Nine principal opportunities were derived from the text comments and responses received during the SWOT analysis, the agreed opportunities are namely:

1. Many recommendations have been made in the course of previous programmes, however, much of this work is between 10 and 15 years old. Opportunity to systematically review all recommendations and to propose up-to-date recommendations based on the findings of the review.
2. To focus E&T in RP on the needs of the current, and future, clinical workforce (including consideration of different areas of practice and different professions and the need to build knowledge, skills, and competences, directly related to benefit-risk communication with patients and the public).
3. To focus E&T in RP on the needs of the current, and future, medical radiation protection researchers (outside the clinical departments and including pre-clinical research).
4. To propose a sustainable and harmonised model for E&T in RP (many past programmes have not succeeded in producing sustainable outcomes).
5. European-level accreditation or endorsement of a recommended, gold standard model of E&T in RP by EURAMED and/or the professional societies EANM, EFOMP, EFRS, ESR, ESTRO.
6. To identify differences in contents and regulations of E&T in RP in EU member states and to propose a European standard for mandatory E&T course contents and certification based on consensus.
7. To stress the importance of well-trained future generations of RP experts with sufficient knowledge, skills, and competences, to cover future needs of E&T.
8. To develop and deliver European-level online training programmes targeting all relevant professional groups to increase accessibility.
9. To develop E&T in RP during the undergraduate course programmes

#### Threats

Threats evidenced from responses during SWOT analysis are summarised in Table 2.

**Table 2:** Summary of the threats evidenced during SWOT analysis

Threats evidenced due to a lack of	
(1) Awareness of E&T in RP and Radiation Application in Medicine (RAM) for health professionals. The importance of E&T training remains present inside a small community or group only	(7) Cohesion between the health and research and the EURATOM communities (EURATOM with low engagement with clinical areas and the health community with low engagement with the EURATOM field)
(2) Time/space or interest by higher education institutions to include E&T in RP and RAM in the curricula of health professions, especially for clinical disciplines	(8) Incentives regarding role development in RP and RAM, leading to health professionals not interested in these topics and in understanding new applications and developments in the field
(3) Translation of real application of E&T in RP and RAM in the clinical setting and inclusion in life-long learning (LLL) for all health professionals involved in the application of ionising radiation. National Health Authorities are only focused on the inclusion of the requirement of E&T in RP and RAM and new technological developments in national legislation	(9) Sufficient importance to E&T in RP and RAM and new technological achievements by national scientific and professional societies which do not attach appropriate importance and or do not include them in consistently.
(4) Importance placed upon the need for E&T in RP by clinical researchers who include medical imaging procedures in their studies (5) Awareness by hospital managers of E&T in RP and RAM importance	(10) Quality control of published document as outputs of previous EU-funded projects with social media and self-learning tools play an increasing role among health professionals. The low impact of E&T in RP and RAM documents increases the potential for sub-optimal information to be challenged
(6) Holistic approach to radiation protection education. Considering that all EU projects until now were focused on/oriented to E&T of Radiation Protection Officer (RPO), Radiation Protection Expert (RPE) and Medical Physics Expert (MPE), the health professionals’ community has the impression that E&T in RP and RAM is only relevant for those group	

### Tows analysis to propose actions to maximise strengths and opportunities to minimise weakness and threats

From the TOWS analysis a list of actions to be developed have been defined and are summarised in Tables 3 and Tables 4 and 5.

**Table 3:** Summary of actions to use strengths to maximise opportunities and minimise threats

	Actions to use strengths to maximise opportunities	Actions to use strengths to minimise threats
Existing structures and training	(1) To systematically review previous recommendations and their impact & implementation (engaging individuals with experience from previous programmes)	
RP training needs assessment & E&T model(s) development	(1) To use MEDRAPET as a basis to formulate and implement a European standard for mandatory E&T IN RP courses and certification (face to face and online learning) based on consensus to meet the needs of the various professional groups (2) To combine experience and learnings from European radiation protection courses & state of the art research with training needs analysis to produce accredited online education programmes (including high quality authentic simulation) accessible to clinicians and researchers alike. Trainer needs to be addressed; including continuous education for the trainers	(1) To use the recent SRAs in order to promote the inclusion of RP and RAM in all clinical research involving radiology, nuclear medicine and radiation oncology (2) To align the SRA for RP and RAM in the medical field with the EURATOM community
E&T dissemination/ harmonisation/ accreditation	(1) To encourage collaboration between regulators, higher education institutes and professional societies to ensure existing regulations (EURATOM) regarding education and training are implemented in member states and follow a consistent standard (2) To promote professional exchange between member states with different standards of RP (3) To use existing networks and collaborations to create a European-level accreditation / endorsement of E&T in RP, supported by regulators	(1) To develop strategies bringing together national authorities, educational institutions, professional societies, safety campaigns and manufacturers to create awareness for the need of harmonisation of procedures involving RAM (2) To use existing EU guidance documents on E&T in RP and RAM as a tool to engage/empower national professional societies of health professionals to achieve implementation of LLL programs in RP and RAM (3) To disseminate the outputs of E&T in RP and RAM elements of EU projects to all relevant stakeholders (4) To increase the cooperation between EURAMED and the existing European networks related to RP, as a strategy to create awareness amongst the stakeholders for the importance of RP and RAM
Financial Supports		(1) To allocate part of the financial support of the E&T in RP and RAM projects to promote and disseminate the results amongst the health professionals’ communities

**Table 4:** Themed opportunities and actions to minimise weaknesses and to minimise weaknesses to avoid threats

Opportunity theme	Actions of opportunities to minimise weaknesses	Actions minimise weakness to avoid threat
Awareness and prioritisation at a nation/global level	(1) To identify differences in content and regulations of E&T in RP in EU member states and to propose a consensus European standard for mandatory E&T course contents and certification, recognised and endorsed by HERCA to better facilitate national implementation (2) To achieve European-level accreditation of curricula and certification of individuals and overcome the national / political challenge of accepting European-level recommendations or qualifications on radiation protection E&T	(1) To standardise training requirements across all member states of the EU (2) To develop a hands-on training program through healthcare facilities following national or European guidelines (3) To develop well-structured awareness campaigns at European and national levels about the importance of E&T in RP and RAM, through the medical professional societies
Awareness and prioritisation by healthcare professional groups and researchers	(1) To improve benefit-risk communication across professional groups and directed at those outside the radiology, nuclear medicine, and radiotherapy departments and at the general public (2) To develop E&T in RP courses focused on clinical needs of the clinical workforce, with input of national and European professional organisations on the ‘model’ design and delivery (3) To develop E&T in RP courses focused on clinical needs and also on non-clinician researchers needs must follow a harmonised model and must consider online approaches to increase accessibility, as well as a modular approach	(1) To stimulate the development of a E&T in RP and RAM guidance document as a collaborative effort by national regulators and professional societies (2) To develop accredited E&T in RP and RAM courses at EU level (3) To develop a profession-specific training program through professional societies following national or European guidelines To develop strategies to bring other health professionals and researchers into the “Radiation Protection interest group”

**Table 5:** Use opportunities to minimise weaknesses and actions to minimise weaknesses to avoid threats

	Actions of opportunities to minimise weaknesses	Actions minimise weakness to avoid threat
Awareness prioritisation by managers and educational institutions	(1) To review existing E&T in RP literature. Plan future design, development, delivery, and evaluation of E&T in RP offerings to be framed within an educational research approach to add to the body of evidence (2) To review all recommendations / listed resources (learning materials and equipment or software to facilitate E&T) and propose up-to-date resource recommendations within a detailed the harmonised model for E&T in RP (3) To develop a suite of ‘bite-sized’ topics within a more extensive model for E&T in RP; to include online offerings, to increase accessibility, and a modular approach (also makes content updates easier) (4) To implement a harmonised model for E&T in RP, including online ‘train the trainers’ courses and stress the importance of education and availability of future generations of radiation protection experts with sufficient knowledge, skills, and competences, to cover future needs of E&T (5) To implement a detailed, harmonised, model for E&T in RP and communication of same to target specific audiences, with pre-reading materials available as required (6) To implement a harmonised model for E&T in RP which describes a blended approach to delivery, including taking online / digital approaches for virtual ‘hands-on’	(1) To develop innovative and accessible education and training programs through higher education institutions in cooperation with healthcare facilities and other relevant stakeholders (2) To cover multiple learning objectives on a specific topic (e.g. dose reduction strategies in CT) through centres of excellence (3) To create an EU network of a multidisciplinary team of experts to “train the trainers” (4) To develop short duration courses focused on 1 key objective (e.g. how to measure dose output in CBCT) (5) To create, and maintain up-to-date, a centralised peer reviewed database of E&T in RP and RAM resources

## Discussion

To our knowledge this is the first TOWS analysis on the SWOT results/impact of E&T in RP aspects developed under previous EU framework programmes and EU-funded projects. Despite the success of the EuroSafe Imaging initiative, which is now in its sixth year of active engagement of its’ call for action to achieve international radiation protection goals this study has identified that there is a significant path ahead to achieve delivery, harmonisation and accreditation of radiation protection training across all stakeholders [14–16]. Extensive literature has been published in recent years which evidences the efforts of European collaborations and the critical work of medical physics and in the achievement of optimal radiation protection practices [17]. Despite this extensive evidence literature base, our SWOT analysis and TOWS findings have identified that there is a need to prioritise resources and efforts to address the weaknesses and threats which have been identified through this study. To facilitate discussion of our findings four areas of consideration are presented.

### Developing training material and trainers

The development of training material and trainers in a manner which is sustainable and facilitates continual refreshing to match technology advances is identified as core consideration for strategic framework planning [18]. Whilst a significant portion of curriculum planning has already been prepared through the work of the MEDRAPET project the sustainable development of training materials is now required. This process needs to align to the continual advances in medical imaging technology and their subsequent radiation protection considerations. Currently, no single training network exists, therefore, it is timely for the stakeholders to consider how a truly holistic approach to radiation protection training could be launched for the benefit all professional disciplines and meet specific professional needs, as identified in this work (Table 2). The Framework to facilitate a truly pan European training network needs to consider how such a process could function at a European, national and local clinical level.

In recent years our access to digital education technologies, has substantially increased, particularly so during the COVID-19 pandemic [19–21]. Educators across multiple professional disciplines have managed this change in teaching practice and have thrived within the online learning environment [22, 23]. The SWOT findings highlight the importance of short online learning objects (Table 4b), this form of learning is promoted across teaching and learning research and the facility for efficient updating is of extreme importance in medical imaging [24]. The willingness of learners to participate in online learning in recent literature is also extremely encouraging and an important consideration when developing material [25–27]. The potential for teaching delivery by a multidisciplinary group of experts at a European level, complimented by nationally supported local teaching, could in some part address the lack of suitably qualified trainers across Europe as identified in TOWS analysis as well as ensuring quality of content [28] The software and technologies used in education now incorporate greater intractability and the use of 2D, 3D and augmented reality software options, which can engage learners more effectivity than some traditional teaching pedagogies and should be incorporated in proposed training frameworks [29].

### Developing “buy-in”

To achieve successful radiation protection training goals, it is essential that those administering ionising radiation in the clinical, research or industry environments see the benefit of training and possess a desire to train. Our findings highlight the importance of meeting profession-specific needs and a current lack of priority placed upon the importance of radiation protection training across professional disciplines, researchers, education institutions, national and European bodies (Table 2). In the most recent European investigation of radiographer radiation protection education in the IAEA, substantial differences in duration and quality of training were highlighted [30, 31]. The literature also highlights similar concerns related to radiation therapy training and the need to improve radiation safety awareness [32]. Similarly, Walsh et.al (2019) identified a low baseline radiation safety knowledge for participating orthopaedic surgeons and trainees. These professionals are frontline workers administering ionising radiation daily and exemplify the need to consider tailored training in addition to core principles. The study highlighted that until now all EU projects have been focussed upon the training of E&T of Radiation Protection Officer (RPO), Radiation Protection Expert (RPE) and Medical Physics Expert (MPE) therefore it is understandable how the health professionals’ community may perceive a lack of professional relevance, and this must be addressed. However there remains a lack of suitably qualified staff to assist in training, predominantly those with a medical physics background, but also from across the stakeholder professions who would be required to assist with training a truly holistic training programme [33, 34].

The challenges ahead should not be underestimated, the impact of the COVID-19 pandemic has further impacted staff resources in both clinical and academic environments, in addition to the increased volume of articles related to professional burnout and the lack of funding for strategic staff contingency planning across healthcare disciplines published pre-COVID-19 [35–37]. In our analysis of how to maximise opportunities and minimise threats, the matter of financial support was identified with the suggestion of using finances to promote training developments to professionals’ communities; however, literature would indicate the need for increased staff resources to facilitate training time and training material development which incorporates realistic budgeting as core to the success of the proposed framework documentation [38, 39].

In addition to the development of training material, we must also consider training the trainers. Higher education institutions are well placed to assist with, considering their experience in teaching and learning pedagogies and access to a broad array of teaching technologies, experienced teachers and education technologists and their incorporation in the EuRnR Framework documentation is essential. To ensure trainers themselves are effective in the clinical, research, industry, and academic environments, it is essential that they themselves are “trained” to a high standard. This is reiterated across several sections of this study aligned to appropriate financial and staff resourcing.

Our findings also identify the need to improve the importance upon which professionals, managers and national agencies place on radiation protection training. The critical role that regulators have in ensuring training programmes are completed is clearly identified in the TOWS analysis (Table 3). EuRnR framework development must specify how regulators and national governments should engage and clarity is required in relation to EuRnR expectations of regulator collaboration. To achieve success the national regulators in turn need to consider how they can influence greater cohesion between the health and research and the EURATOM communities nationally under the single umbrella of radiation protection training in their efforts to protect the public [40].

### Embedding RP training and accreditation

Once training materials and networks are developed our study has identified the importance of determining how a radiation protection network on a European scale, which has national and local on-site activity, could be embedded as mandatory practice by employers and regulators and potentially also have employment incentives potentially attached (Table 2). Furthermore, the issue of accreditation, quality control and training oversight was a recurring item of the SWOT investigation and TOWS analysis. The EuRnR strategic framework documentation will need to consider how accreditation processes are recognised by national regulators and professional groups as national legislation may demand national accreditation of training and not be legally permitted to recognise a pan European accreditation process. How this matter is managed is critical, as mandatory compliance with regulation agencies linked to government bodies is important in the achievement of sufficient funding for the development of a successful radiation protection training network and one which is mandated as a continued professional development requirement.

### Strategic resource planning of RP training

As stated within the discussion at different junctures as with any training programme, the cost of development and implementation requires consideration: from the funding of time and expertise to develop teaching material, hosting virtual/online learning environments, teaching material delivery, trainer costs, academic and clinical site costs, accreditation costs and resourcing of quality review processes. All these items are significant and will differ across nations and locally across clinical, academic and industrial sites and how this can be managed requires intensive discussion and must be clearly and realistically addressed in the proposed EuRnR strategic framework documentation to be completed by this project.

### Limitations

In the past, most EU-funded programmes and projects were profession specific or not directly related to medical procedures. Nevertheless, considering the composition of the EuRnR consortium, the group brings together experts in RP from the most relevant health professions and researchers involved in the clinical use of ionising radiation (e.g. Radiologists, Nuclear Medicine Physicians, Radiographers, Medical Physicists, Radiation Oncologists), it was possible to minimise the described limitation, through several multi-professional group meetings, as a strategy to obtain consensus regarding the SWOT analysis and the respective TOWS actions. Future consideration does need to incorporate the expertise of E&T scientists as the EuRnR framework documentation is progressed.

## Conclusion

E&T in RP is of paramount importance for health professionals and researchers to acquire and develop knowledge, skills and competences in the field of RP to protect patients and staff from the dangers arising from the exposure to ionising radiation. Although several projects have been developed in the past years related to E&T in RP, the SWOT analysis showed a clear lack of real and effective implementation of RP principles in daily practice and therefore there is a critical opportunity under EuRnR to define and set a new momentum, through an objective and dedicated action plan for E&T in RP in Europe. EuRnR Strategic planning documentation needs to consider processes at European, national and local levels and incorporate the multiple factors identified in this SWOT investigation and TOWS analysis. To achieve success, governance structures and strong leadership are key as is the full exploitation of existing resources however equally, appropriate financial support is essential to permit our professions to work collaboratively to achieve a pan European radiation protection training network which is sustainable and accredited across multiple national domains.

## Data Availability

The survey tool and raw data are securely stored by the research team within the EuRnR: EURAMED rocc-n-roll project and will be available until the close of the EU-funded project. The data sets generated and analysed during this current study are available from the corresponding author on reasonable request.

## Abbreviations

CIRSE: Cardiovascular and Interventional Radiological Society of Europe
CONCERT: European Joint Programme for the Integration of Radiation Protection Research
COVID-19: Coronavirus disease
DoReMi: Low Dose Research towards Multidisciplinary Integration
EANM: European Association of Nuclear Medicine
EFOMP: European Federation of Organisations for Medical Physics
EFRS: European Federation of Radiographer Societies
ENEN +: European Nuclear Education Network
ENETRAP: European Network on Education and Training in Radiological Protection
ESR: European Society of Radiology
ESTRO: European Society for Radiotherapy and Oncology
EU: European Union
EURAMED: European Alliance for Medical Radiation Protection Research
EuRnR: EURAMED rocc-n-roll project
HERCA: Heads of the European Radiation Protection Authorities
IAEA: International Atomic Energy Agency
ICRP: International Commission on Radiological Protection
MEDRAPET: Medical Radiation Protection Education and Training
MELODI: Multidisciplinary European Low Dose Initiative
EURADOS: European Radiation Dosimetry Group
MPE: Medical Physics Expert
OPERA: Open Project for the European Radiation Research Area
RP: Radiation protection
RPE: Radiation protection expert
RPO: Radiation protection officer
SRA: Strategic research agenda
SWOT: Strengths, weaknesses, opportunities and threats
TOWS: Threats, opportunities, weaknesses and strengths
WHO: World Health Organisation
